# Detection and Viability of *Lactococcus lactis* throughout Cheese Ripening

**DOI:** 10.1371/journal.pone.0114280

**Published:** 2014-12-15

**Authors:** Marianna Ruggirello, Paola Dolci, Luca Cocolin

**Affiliations:** Dipartimento di Scienze Agrarie, Forestali e Alimentari, Università degli Studi di Torino, Grugliasco, Italy; Institut Pasteur de Lille, France

## Abstract

Recent evidences highlighted the presence of *Lactococcus lactis* during late cheese ripening. For this reason, the role of this microorganism, well known as dairy starter, should be reconsidered throughout cheese manufacturing and ripening. Thus, the main objective of this study was to develop a RT-qPCR protocol for the detection, quantification and determination of the viability of *L. lactis* in ripened cheese samples by direct analysis of microbial nucleic acids. Standard curves were constructed for the specific quantification of *L. lactis* in cheese matrices and good results in terms of selectivity, correlation coefficient and efficiency were obtained. Thirty-three ripened cheeses were analyzed and, on the basis of RNA analysis, twelve samples showed 10^6^ to 10^8^ CFU of *L. lactis* per gram of product, thirteen from 10^3^ to 10^5^ CFU/g, and in eight cheeses, *L. lactis* was not detected. Traditional plating on M17 medium led to loads ranging from 10^5^ to 10^9^ CFU/g, including the cheese samples where no *L. lactis* was found by RT-qPCR. From these cheeses, none of the colonies isolated on M17 medium was identified as *L. lactis* species. These data could be interpreted as a lack of selectivity of M17 medium where colony growth is not always related to lactococcal species. At the same time, the absence or low abundance of *L. lactis* isolates on M17 medium from cheese where *L. lactis* was detected by RT-qPCR support the hypothesis that *L. lactis* starter populations are mainly present in viable but not culturable state during ripening and, for this reason, culture-dependent methods have to be supplemented with direct analysis of cheese.

## Introduction

The study of microbial ecology associated with dairy fermentations is fundamental to understand the bases of important traits of dairy products. Traditionally, microbial dynamics in dairy fermentations have been studied with methods based on cultivation on selective media followed by phenotypic and/or molecular characterization. These approaches highlighted the role and activity, in cheese manufacturing and ripening, of two microbial groups: starter lactic acid bacteria (LAB)(mainly *Lactococcus lactis*, *Streptococcus thermophilus* and *Lactobacillus* spp.), with primary function of producing sufficient lactic acid during cheese manufacturing to reduce the pH of the milk; and non-starter LAB (NSLAB) (mainly *Lactobacillus* spp., *Pediococcus* spp., *Enterococcus* spp. and *Leuconostoc* spp.), generally adventitious contaminants which grow later, during cheese ripening, with an impact on flavour development [1].

In the last years, approaches to study microorganisms in dairy products have undoubtedly changed. Culture-dependent approaches have shown limitations in terms of recovery rate, mainly related to the lack of knowledge of the real conditions under which most of bacteria are growing in their natural habitat, and the difficulty to develop media for cultivation accurately resembling these conditions [2], [3]. Thus, the cultivable populations may not totally represent the community, and the actual microbial diversity could be misinterpreted [4]. For these reasons, the trend is now towards the use of culture-independent methods because they are believed to overcome problems associated with selective cultivation and isolation of bacteria from dairy samples. The development of these techniques has revolutionized microbial ecology and their application in cheese microbiology is leading to new insights. In particular, in recent studies, some authors have hypothesized the presence of metabolically active starter populations throughout cheese ripening [5]–[8]. In particular, Rantsiou et al. [6] showed the presence of alive population of *S. thermophilus* and *Lactococcus* spp. in ripened Feta cheese by using the culture-independent technique FISH (Fluorescence in situ hybridization). Later, Dolci et al. [8] found metabolically active population of *L. lactis* in ripened Castelmagno PDO cheese by RT-PCR-DGGE (Denaturing Gradient Gel Electrophoresis). Thus, it should be considered the possibility that starter populations are in a viable but not culturable (VNC) state during cheese ripening and, for this reasons, culture-dependent methods are not able to highlight their presence. In support of this hypothesis, VNC state has been described for *L. lactis* as a physiological response to carbohydrate starvation [9], a situation that characterizes a cheese after fermentation [10]. The main objective of this study was to develop a RT-qPCR protocol for the detection, quantification and determination of the viability of *L. lactis* in ripened cheese samples by direct analysis of microbial nucleic acids. In parallel, a culture-dependent approach was carried out in order to investigate, in ripening conditions, the presence/absence of *L. lactis* cells able to grow on selective medium.

## Materials and Methods

### 1 Optimization of quantitative PCR protocol for the detection of *L. lactis*

#### 1.1 Primer design

The housekeeping gene *tuf* [7], encoding for the elongation factor tu [11], was chosen as target for the design of *L. lactis* specific primers. *Tuf* gene sequences of *L. lactis* subsp. *lactis*, subsp. *cremoris* and of other LAB species, commonly found in dairy products, were retrieved in GenBank (http://www.ncbi.nlm.nih.gov/genbank/)(Table 1) and aligned using the software Clustal W2, Multiple Sequence Alignment (http://www.ebi.ac.uk/Tools/msa/clustalw2/). For primer design, Primer3 software (http://primer3.ut.ee/) was employed. Finally, the BLAST search tool (Basic Local Alignment Search Tool, http://blast.ncbi.nlm.nih.gov/Blast.cgi) was used to check the *in silico* specificity of the selected primer pair Tuf2 forward (Tuf2f) (5′-TGA ACC ACA ATG GGT TGC TA-3′) and Tuf2 reverse (Tuf2r) (5′-TCG ACT GGA AGA AGG AGT GG -3′).

**Table 1:** Accession number of the sequences used for the design of *L. lactis* Tuf2 specific primers and the species of LAB to which they belong.

GenBank accession number	LAB species
CP000033.3	*Lactobacillus acidophilus* NCFM
CP000416.1	*Lactobacillus brevis* ATCC 367
CP000423.1	*Lactobacillus casei* ATCC 334
FM177140.1	*Lactobacillus casei* BL23
CR954253.1	*Lactobacillus delbrueckii* subsp. *bulgaricus* ATCC 11842
CP000412.1	*Lactobacillus delbrueckii* subsp. *bulgaricus* ATCC BAA-365
AM295061.1	*Lactobacillus fermentum* subsp. *cellobiosus* DSM 20055
FJ825125.1	*Lactobacillus helveticus* IMAU30062(C8402)
CP001617.1	*Lactobacillus plantarum* JDM1
AL935263.2	*Lactobacillus plantarum* WCFS1
AM406671.1	*Lactococcus lactis* subsp. *cremoris* MG1363
CP000425.1	*Lactococcus lactis* subsp. *cremoris* SK11
NC002662.1	*Lactococcus lactis* subsp. *lactis* Il1403
CP000422.1	*Pediococcus pentosaceus* ATCC 25745
CP000046.1	*Staphylococcus aureus* subsp. *aureus* COL
CP000255.1	*Staphylococcus aureus* subsp. *aureus* USA300_FPR3757
CP000023.1	*Streptococcus thermophilus* LMG 18311
CP000024.1	*Streptococcus thermophilus* CNRZ1066
CP000419.1	*Streptococcus thermophilus* LMD-9
AE009948.1	*Streptococcus agalactiae* 2603V/R
AL766847.1	*Streptococcus agalactiae* NEM316
CP000114.1	*Streptococcus agalactiae* A909
AE016830.1	*Enterococcus faecalis* V583
CP003351.1	*Enterococcus faecium* Aus0004

#### 1.2 Bacterial strains and DNA extraction

In order to optimize the amplification conditions and to assess the specificity of the qPCR protocol for *L. lactis*, the primers Tuf2f and Tuf2r, synthesized by Sigma-Aldrich (Milan, Italy), were used on target and non-target LAB commonly found in dairy products (Table 2). The bacterial strains were grown overnight at 37°C in M17 broth supplemented with lactose (5 g/L) (lactococci, enterococci and streptococci)(Oxoid, Milan, Italy) and in MRS broth (lactobacilli)(Oxoid). In addition, two strains of *Staphylococcus aureus* were cultivated in BHI broth (Oxoid) at 37°C. One millilitre of each culture was centrifuged at 20,844 g for 5 min, the supernatant discarded and the resulting pellet submitted to DNA extraction according to the protocol reported from Cocolin et al. [12]. DNA yield and quality were determined with the NanoDrop ND-1000 spectrophotometer (NanoDrop Technologies, Wilmington, DE, USA).

**Table 2:** Bacterial strains commonly found in dairy products and used in this study to optimize and assess the specificity of the qPCR protocol for *L. lactis*.

Bacterial species	Strain code^a^
*Enterococcus faecalis*	BF 14^D^
*Enterococcus faecalis*	551^D^
*Enterococcus faecalis*	30FE3^D^
*Enterococcus faecalis*	30FE4^D^
*Enterococcus faecium*	ATCC 19434T^N^
*Enterococcus faecium*	37FE2^D^
*Enterococcus faecium*	37FE3^D^
*Enterococcus faecium*	571^D^
*Enterococcus faecium*	Ma F115^D^
*Leuconostoc mesenteroides*	Lm 24^N^
*Lactobacillus brevis*	CNRZ 734^N^
*Lactobacillus casei*	F30Bi4^D^
*Lactobacillus casei*	F150AeM5^D^
*Lactobacillus casei shirota*	F17MAR^D^
*Lactobacillus delbrueckii*	c 91^D^
*Lactobacillus delbrueckii*	c 91^D^
*Lactobacillus delbrueckii*	c 92^D^
*Lactobacillus delbrueckii*	c 93^D^
*Lactobacillus delbrueckii*	c 94^D^
*Lactobacillus helveticus*	m 80^N^
*Lactobacillus helveticus*	m 81^D^
*Lactobacillus paracasei*	P 95^D^
*Lactobacillus paracasei*	P 96^D^
*Lactobacillus paracasei*	DSM 5622T^N^
*Lactobacillus plantarum*	110 ag^D^
*Lactobacillus plantarum*	a 810^D^
*Lactobacillus plantarum*	Lab7^D^
*Lactobacillus plantarum*	ATCC 4008T^N^
*Lactococcus garvieae*	DSM 20684^N^
*Lactococcus lactis* subsp. *cremoris*	Fc95^D^
*Lactococcus lactis* subsp. *cremoris*	Fc96^D^
*Lactococcus lactis* subsp. *lactis*	39FL4^D^
*Lactococcus lactis* subsp. *lactis*	39FL3^D^
*Lactococcus lactis* subsp. *lactis*	39FL2^D^
*Lactococcus lactis* subsp. *lactis*	39FL1^D^
*Lactococcus lactis* subsp. *lactis*	DSM 20481T^N^
*Staphylococcus aureus*	661^D^
*Staphylococcus aureus*	662^D^
*Streptococcus thermophilus*	LCM 3^D^
*Streptococcus thermophilus*	6C6^D^
*Streptococcus thermophilus*	7C6^D^
*Streptococcus thermophilus*	8C6^D^
*Streptococcus thermophilus*	4C10^D^
*Streptococcus thermophilus*	4C16^D^
*Streptococcus thermophilus*	6C16^D^
*Streptococcus thermophilus*	10C7^D^
*Streptococcus thermophilus*	DSM 20617T^N^
*Streptococcus thermophilus*	DSM 20617T^N^

a D: strains from DISAFA Collection (University of Turin, Italy) isolated from dairy products; N: strains from International Collections.

#### 1.3 Amplification conditions

One-hundred nanograms of DNA extracted from each strain was used in qPCR in order to optimize the protocol. Different primer concentrations and annealing temperatures were tested to determine the most selective and efficient amplification conditions. The best results were obtained with Tuf2f and Tuf2r added at 400 nM and 50 nM, respectively, and using the SsoAdvanced^TM^ SYBR@ Green Supermix (Bio-Rad, Hercules, CA, USA) in a final volume of 20 µL. Quantitative PCR assays were performed in Chromo4 Real Time PCR Detection System (Bio-Rad) with the software MJ OpticonMonitor version 3.1. The thermal cycle conditions were optimized as follows: an initial denaturation at 98°C for 2 min, 40 cycles at 95°C for 5 sec and 68.7°C for 30 sec, where the stringent annealing temperature value, and the cumulative annealing and extension steps were chosen to increase the selectivity of the protocol to the microorganism target *L. lactis*.

### 2 Construction of *L. lactis* standard curves

#### 2.1 Standard curves from pure culture

Standard curves were constructed from ten-fold serial dilutions of an overnight pure culture of *L. lactis* subsp. *lactis* 39FL4 (DISAFA collection). One millilitre of each dilution was submitted to RNA extraction using the MasterPureTM Complete DNA and RNA Purification Kit (Epicentre, Madison, WI, USA) following the manufacturer's instructions, with the addition of lysozyme (50 mg/mL, Sigma, Milan, Italy) to improve cell wall lyses. Three microliters of TURBO - DNase (Ambion, Milan, Italy) were added to digest the DNA in the RNA samples, with an incubation of 3 h at 37°C. The absence of residual DNA, in the RNA samples, was checked by qPCR and, subsequently, reverse transcription (RT) was performed by using the M-MLV Reverse Transcriptase kit (Promega, Milan, Italy). RT was performed as follows: 5 µL of RNA were mixed with 1 µL of TUF2r primer (100 µM) in a reaction volume of 10 µl by addition of ultrapure water. The mix was treated at 75°C for 5 min for RNA denaturation and immediately placed on ice for 10 min. Five microliters of M-MLV RT Buffer (1×), 5 µl of dNTPs (10 µM each), 1 µl of M-MLV Reverse Transcriptase (8 U/µL) and 0.6 µL of RNasin ribonuclease inhibitor (20 U/µL) were added to the mix for a final volume of 25 µl by addition of ultrapure water. RT reaction was carried out at 42°C for 1 h in a Biorad DNA Engine thermal cycler (Bio-Rad) and the cDNA samples stored at −30°C. cDNA was subjected to qPCR, according to the amplification conditions described in paragraph 1.3. *S*tandard curve was constructed by plotting the Ct values against the colony forming units (CFU)/mL of the overnight *L. lactis* subsp. *lactis* 39FL4 culture, evaluated by traditional plating on M17 agar supplemented with lactose (5 g/L)(Oxoid). The reactions were carried out in triplicate and mean values were considered for standard curve construction. The correlation coefficients (*R^2^*) and the efficiency (*E*) of the amplifications were calculated according to Rutledge and Cote [13].

#### 2.2 Standard curves from inoculated cheese matrix and evaluation of L. lactis detection limit

Ten grams of grated cheese (Biraghi s.p.a., Cuneo, Italy) were contaminated with 10 mL of ten-fold serial dilutions of an overnight pure culture of *L. lactis* subsp. *lactis* 39FL4 and added of 30 mL of sterile Ringer solution (Oxoid). The samples were homogenized in a Stomacher (Interscience, Rockland, MA, USA) for 30 sec and 1 mL of each sample was subjected to RNA extraction by using the MasterPureTM Complete DNA and RNA Purification Kit (Epicentre). Ribonucleic acid samples were treated as reported above (paragraph 2.1) and each cDNA sample was submitted to qPCR. Standard curve was constructed by plotting the Ct values against CFU/g, evaluated by traditional plating on M17 agar supplemented with lactose (5 g/L), of the grated cheese inoculated with the overnight *L. lactis* subsp. *lactis* 39FL4 culture. The reactions were carried out in triplicate and mean values were considered for standard curve construction. The *R^2^* and *E* of the amplifications were calculated according to Rutledge and Cote [13].

The limit of detection of *L. lactis* was evaluated by RT-qPCR in grated cheese specifically inoculated with 5, 10, 50, 100, 1000 CFU/g of the microorganism. Commercial grated cheese was checked for the absence of lactococci before use.

### 3 Detection of *L. lactis* in commercial cheeses by culture-dependent and -independent methods

Thirty-three ripened cheeses (Table 3) were purchased from the Italian market. The cheeses were available for sale in a pre-packed format with an approximate weight of 100 g each.

**Table 3:** Quantification of *L. lactis* in the ripened cheeses analyzed in this study and comparison of the results obtained by using culture-independent and -dependent approaches.

	Culture-independent approach		Culture-dependent approach	
Cheese sample and ripening time (day)	RT-qPCR	M17 agar plating	Molecular identification of the isolates	
	LOG CFU/g cheese ± SD	LOG CFU/g ± SD	*L. lactis* RSA profile	Positive to HIS-PCR
Asiago d'allevo PDO 120 d	*	8.32±0.73	0/12	0/0
Asiago PDO 90 d	7.13±0.02	6.94±0.43	2/12	2/2
Bra tenero PDO 45 d	*	8.41±0.32	0/12	0/0
Castelmagno 90 d	5.34±0.03	6.58±0.24	4/12	0/4
Castelmagno PDO 180 d	6.17±0.05	5.40±0.43	2/12	1/2
Fontal 60 d	5.06±0.03	5.48±0.32	2/12	0/2
Fontina PDO 120 d	3.82±0.01	6.86±0.24	3/12	0/3
Fontina PDO 120 d	5.39±0.06	8.36±0.14	1/12	0/1
Fontina PDO 120 d	6.11±0.06	8.59±0.75	4/12	0/4
Fontina PDO 120 d	5.50±0.01	7.33±0.21	3/12	0/3
Fontina PDO 120 d	3.78±0.02	6.38±0.56	1/12	0/1
Pecorino di Gallura	6.97±0.04	9.48±0.42	4/12	0/4
Fiore Sardo 120 d	6.29±0.02	7.57±0.12	3/12	0/3
Pecorino fioretto 120 d	7.48±0.07	7.79±0.66	7/12	7/7
Pecorino pastore 90 d	*	5.70±0.55	1/12	0/1
Pecorino romano 120 d	5.56±0.04	6.88±0.46	5/12	0/5
Pecorino sardo PDO 120 d	4.96±0.07	7.76±0.78	2/12	0/2
Pecorino toscano PDO 90 d	7.58±0.04	6.51±0.54	0/12	0/0
Raschera 60 d	*	8.23±0.23	0/12	0/0
Raschera PDO 90 d	3.76±0.03	6.40±0.64	2/12	0/2
Raschera PDO 90 d	5.24±0.02	8.01±0.22	3/12	0/3
Raschera PDO 60 d	*	9.52±0.19	2/12	0/2
Toma d'Oropa 45 d	*	7.77±0.45	4/12	0/4
Toma di Bra 45 d	*	9.16±0.33	2/12	0/2
Toma di capra 60 d	8.47±0.01	8.59±0.56	12/12	6/12
Toma di Lanzo 60 d	7.06±0.01	8.15±0.16	6/12	1/6
Toma di Lanzo 60 d	4.55±0.02	8.88±0.24	3/12	0/3
Toma di Lanzo 60 d	4.82±0.02	8.92±0.21	4/12	3/4
Toma di Lanzo 60 d	6.90±0.02	8.60±0.31	5/12	1/5
Toma piemontese PDO 60 d	3.72±0.10	8.38±0.77	4/12	0/4
Toma piemontese PDO 60 d	*	9.19±0.13	3/12	0/3
Toma piemontese PDO 60 d	7.68±0.00	8.95±0.12	7/12	3/7
Toma piemontese PDO 60 d	7.40±0.02	7.45±0.48	4/12	0/4

*below quantification limit.

SD: standard deviation.

#### 3.1 Detection of L. lactis in cheese samples by RT-qPCR assays

Ten grams of each cheese were homogenized in 40 mL of sterile Ringer solution (Oxoid) in a Stomacher (Interscience) for 1 min, and 1 mL of the homogenized suspension, in duplicate, was centrifuged at 20,844 g for 5 min to pellet the microbial cells for subsequent RNA extraction. The MasterPureTM Complete DNA and RNA Purification Kit (Epicentre) was used for nucleic acid extraction as reported above (2.1). Ribonucleic acid samples were treated with DNase and checked for the efficiency of the treatment, as reported in paragraph 2.1. RT was performed by using the M-MLV Reverse Transcriptase kit (Promega) (2.1).

#### 3.2 Detection of L. lactis in cheese samples by traditional plating and species-specific PCR

The cheese samples, homogenized as described above, were serially diluted and plated on M17 agar media (Oxoid) supplemented with lactose (5 g/L), supposed to be selective for lactic acid cocci. The plates were incubated, aerobically, at 37°C for 48 h and, after counting, 12 randomly selected colonies were isolated for each sample. A total of 396 isolates were grown overnight at 37°C in M17 broth (Oxoid) supplemented with lactose (5 g/L). One millilitre of each culture was transferred to a 1.5 mL screw cap tubes containing 0.3 g of glass beads with a diameter of 0.5 mm and centrifuged at 20,844 g for 5 min. The supernatant was discarded and the resulting pellet was stored at −20°C until DNA extraction as previously reported [12].

The identification of *L. lactis* isolates was carried out by combining PCR 16S–23S rRNA gene spacer analysis (RSA) [13] and *L. lactis* specific PCR, based on the amplification of a portion of the histidine biosynthesis operon (His-PCR) [14]. On the basis of RSA electrophoretic patterns, the isolates showing a single band were checked by His-PCR to confirm their belonging to *L. lactis* species.

## Results

### 4 Specificity of the qPCR protocol to *L. lactis* strains

The specificity of the primer couple Tuf2f and Tuf2r was assessed by qPCR using the genomic DNA of the bacterial strains reported in Table 2. The fluorescence signal was detected for the subspecies *L. lactis* subsp. *lactis* and subsp. *cremoris* at Ct values of, on average, 14.37 (±0.12) and 15.69 (±0.31), respectively; strains belonging to species phylogenetically related to *L. lactis* and/or commonly found in dairy products were not amplified within the 40 cycles set in the amplification protocol. The melting curve of the amplicons obtained from qPCR for *L. lactis* strains was studied and a single peak was observed for each reaction; the absence of secondary amplification products confirmed the high specificity of the protocol optimized.

### 5 Standard curves and *L. lactis* detection limit

In order to quantify *L. lactis*, standard curves were constructed both from pure cultures and using grated cheese, simulating a long ripened cheese matrix, inoculated with pure cultures of *L. lactis*. Lactococci were not found in the grated matrices before inoculation by both cultivation on M17 medium (<5 cfu/g) and direct RT-qPCR (<10 cfu/g).

**Figure 1:**
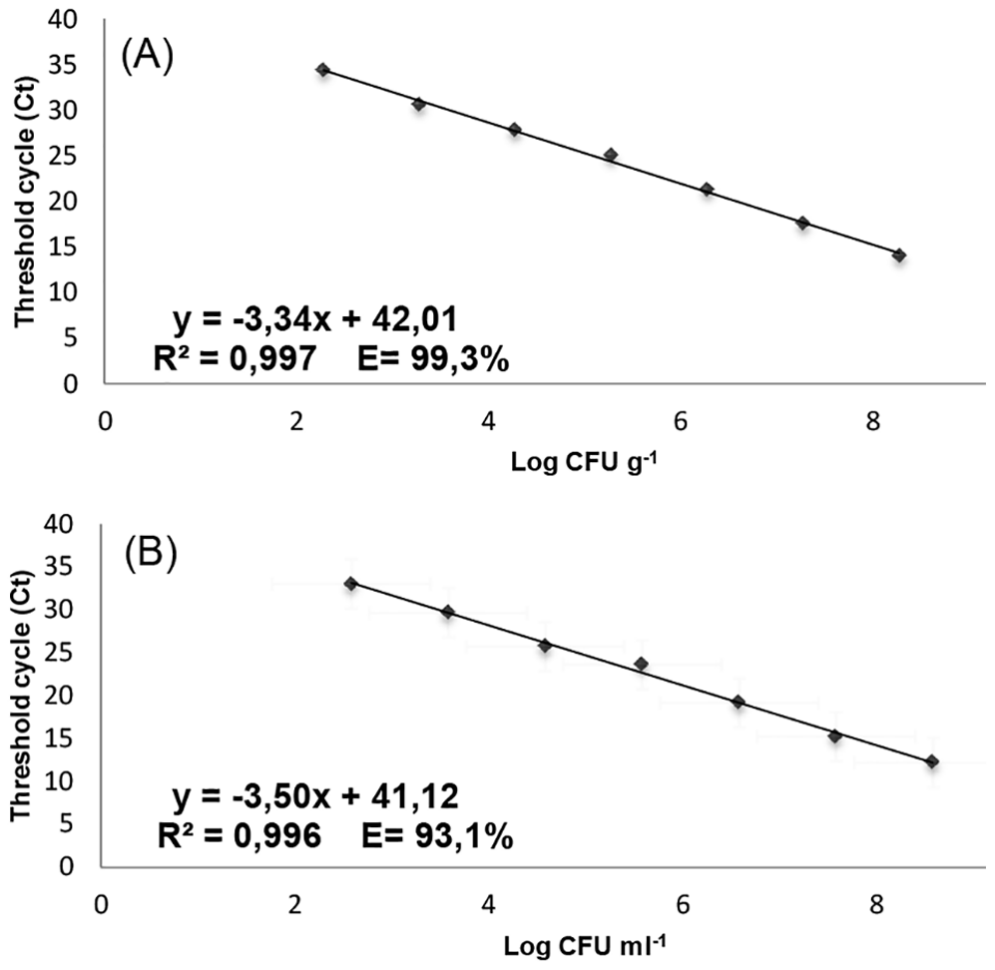
Standard curves obtained by RT-qPCR for *L. lactis* inoculated in cheese matrix (A) and *L. lactis* pure culture (B).

A quantification limit of 10^2^ CFU/mL and CFU/g was obtained for *L. lactis* RNA extracted from pure culture and grated cheese, respectively, and the linearity range was spanning from 10^2^ to 10^8^ CFU/mL or g, covering 6 orders of magnitude. A good linear correlation between the Ct values and *L. lactis* cell loads was obtained for the standard curves constructed, showing *R^2^* values of 0.997 and 0.996 for inoculated cheese and pure cultures, respectively; and *E* values of 99,3% and 93,1% for inoculated cheese and pure cultures, respectively. The standard curves with the relative equation, R^2^ and *E* are reported in Fig. 1. The results obtained in terms of quantification limit, efficiency of amplification and coefficient of correlation derived by three independent experiments. Standard deviations have not been reported in Fig. 1 because of their low values (data not shown).

The limit of detection of *L. lactis* in grated cheese, evaluated by RT-qPCR, corresponded to 10 CFU/g.

### 6 Viability of *L. lactis* in ripened cheeses

#### 6.1 Culture-independent approach

Cheese samples on the market (Table 3) were analyzed to assess the applicability of RT-qPCR protocols for the direct quantification of *L. lactis* in dairy matrices, and to investigate the viability of this microorganism in ripened products.

Twenty-five samples, on a total of thirty-three, resulted positive for the presence of viable cells of *L. lactis*, as detected by RT-qPCR. The interpolation of the Ct values obtained, in the standard curve equation from inoculated cheese (Fig. 1A), resulted in *L. lactis* loads ranging from 10^3^ to 10^8^ CFU/g for RNA analysis (Table 3). The highest values were obtained for some of the Toma, Pecorino and Asiago cheese type samples, with loads higher than 10^7^ CFU/g. In Fontina cheese, *L. lactis* counts ranged from 10^3^ to 10^6^ CFU/g, while in Castelmagno, *L. lactis* was detected with values of 10^5^ and 10^6^ CFU/g. In Raschera, *L. lactis* was found with loads of 10^3^ and 10^5^ CFU/g. On the contrary, eight samples belonging to Asiago, Bra, Pecorino, Raschera and Toma cheese type, were negative for *L. lactis* viability.

#### 6.2 Culture-dependent approach

The cheese samples purchased from the market were subjected to traditional plating on M17 medium and the values ranged from 10^5^ to 10^9^ CFU/g (Table 3). A total of 396 colonies were randomly selected, purified and subjected to molecular identification; 78 isolates showed a RSA electrophoretic profile characterized by an unique band migrating approximately at 390 bp, characteristic of *L. lactis* species [15]. Species-specific His-PCR was necessary to finally establish the taxonomic position of the isolates of which only 24, spread among eight cheese samples (Table 3), resulted to belong to *L. lactis* species.

## Discussion

The importance to monitor cheese microbial populations has been considered by different authors and, now, the literature is rich in papers focused on this topic [16]. In particular, it has been investigated the role of LAB, during the most effective technological phases, when it is important to have certain microbial activities to achieve the expected quality of the final product.

The primary role of starter LAB in cheese is considered a *dogma* in dairy microbiology. They produce high amount of lactic acid, causing milk acidification, and represent a bio-catalytic potential for cheese-ripening reactions, through the liberation of hydrolytic intracellular enzymes following autolysis [1]. Feirtag and McKay [17] first reported this phenomenon for lactococci and associated their autolytic activity to enhanced flavour development in cheese.

As reported in the introduction, recent studies have highlighted the presence, throughout cheese ripening, of alive cells of *L. lactis* by culture-independent techniques based on FISH [6], RT-PCR-DGGE [8] and RT-qPCR [18]. These evidences impose a more careful understanding of the role of *L. lactis*, in the ripening process, not only in terms of autolytic activity, but also as metabolically active cells.

In the present study, we investigated the presence of *L. lactis* populations in different ripened cheeses available on the market. In accordance with Desfossés-Foucault et al. [18] and supporting the first evidences cited above [6], [8], the results confirmed the presence of viable cells of *L. lactis* in cheeses, at the end of the ripening time. Thirty-three cheeses were analyzed. On the basis of RT-qPCR results, twelve samples showed 10^6^ to 10^8^ CFU of *L. lactis* per gram of product, and thirteen from 10^3^ to 10^5^ CFU/g. In eight cheeses, *L. lactis* was not found (*L. lactis* detection limit in grated cheese: 10 CFU/g), thus, the microorganism was considered not involved in the ripening of these products. Traditional plating on M17 medium led to loads ranging from 10^5^ to 10^9^ CFU/g, including cheese samples were no *L. lactis* was found by RT-qPCR. In these cheeses, none of the colonies isolated on M17 medium was identified as *L. lactis*. These data could be interpreted as a lack of selectivity of M17 medium where colony growth is not always related to lactococcal species. Probably, lactococci are able to grow on M17 medium when they are abundant and not stressed, as for example during milk and curd fermentation. Differently, during the ripening process, it is known that NSLAB increase in number and prevail on lactococcal populations, which are often out-competed by the numerically more abundant lactobacilli. Nevertheless, in this work, a few isolates were identified as *L. lactis* by His-PCR. They were obtained from eight cheese samples with loads higher than 10^7^ CFU/g, detected by RT-qPCR, except for two samples characterized by values of 10^4^ and 10^6^ CFU/g. These data could be explained with the relative high abundance of *L. lactis* in these cheeses and, thus, its capability to compete with the rest of microbiota and multiply on synthetic media. On the basis of the results obtained, and as reported by other authors [19], alternative cultural approaches should be better considered. Currently, M17 is the medium mainly used for lactococci cultivation, but new formulations for the isolation of LAB from cheese have been recently studied as, for example, cheese agar [19], which was used to recover minority populations from milk, whey starter and fresh curd of Parmigiano Reggiano, hardly estimable on traditional media. Thus, as future prospective, for a more reliable and effective recovery of lactococci, in particular *L. lactis*, during cheese ripening, the optimization and formulation of specific nutritional conditions should be better investigated.

The absence or low abundance of *L. lactis* isolates on M17 medium, support the thesis hypothesized by other authors [9] that *L. lactis* starter populations are mainly present in VNC state during cheese ripening and, for this reason, culture-dependent methods are not able to detect their presence and have to be complemented with direct analysis in cheese. These considerations can be especially corroborated from the results obtained in eight cheese samples where the difference, in terms of microbial load, between RT-qPCR and plating data, was lower than 10^2^ CFU/g and, thus, the absence of *L. lactis* growth, on M17, could not be justifiable with the prevalence of NSLAB.

For some of the cheeses analysed, experiments were performed in order to “resuscitate” *L. lactis* VNC cells and preliminary results highlighted that different carbon sources, in cultural media, affect differently their growing ability (data not shown); in particular, enrichment in medium with high percentage of glucose (2.5%) seemed to stimulate the attitude of the cells to become culturable again (data not shown). Recent researches were focused on these aspects and highlighted the presence of VNC *L. lactis* cells in ripened cheese products [18], [20]. Differently, Flórez and colleagues [5] found abundance of *L. lactis* isolates on M17 from the analysis of Spanish cheese, but they did not specify the distribution of the isolates among milk, curd and cheese samples. In addition, Desfossés-Foucault et al. [18] recently described that some species may be unable to grow even if they are in a viable and metabolically active state in the food matrix. Thus, culture-independent methods allow to overcome biases associated to the culturing step. The detection of microbial populations from total DNA or RNA extracted directly from food matrices can give a more realistic and reliable “picture” of cheese microbiota.

In order to monitor the presence and viability of *L. lactis* throughout cheese manufacturing and ripening, a highly selective qPCR protocol was optimized. The detection of *L. lactis* with respect to other LAB species, which normally colonize ripened cheeses, was reached by selective primer design on *tuf* gene codyfing a GTP binding protein and widespread in eubacteria genomes [21]. *Tuf* gene has been generally recognized as a housekeeping gene [21]; moreover, its stability was confirmed by studying its expression throughout *L. lactis* growth curve (data not shown).

SYBR green fluorescent chemistry was chosen and good results were obtained, in terms of specificity, correlation coefficient and efficiency, by increasing the stringency of the thermal cycle and using primers in unbalanced concentration. In particular, the high (compared to primer melting temperature) annealing temperature, used in qPCR and RT-qPCR protocols, allowed the specific detection of *L. lactis* and no fluorescent signal was detected when the protocol was applied to the other LAB species. Thus, *tuf* gene represented a suitable target for the specific detection and quantification of *L. lactis* as also highlighted by other authors [20]. Moreover, the efficiency of the protocols was improved by the choice of nucleic acid extraction protocols specifically designed for the treatment of fatty matrices [22], highlighting, once again, how this step heavily influence the performance of the subsequent amplification. The high quality of the extracted RNA and the set amplification conditions allowed to obtain standard curves with a good linearity range covering 6 orders of magnitude, from 10^2^ to 10^8^ CFU/g.

Other authors optimized qPCR protocols to detect *L. lactis* in milk [3], under simulated conditions of cheese manufacture [23], in ultrafiltered milk cheese models [7], [24] and in the manufacturing of raw milk soft cheeses [20]. The only study dealing with the monitoring of active population of *L. lactis* in cheese ripening by RT-qPCR has been focused on Cheddar cheese [18], for which undefined starter cultures containing *L. lactis* subsp. *lactis* and *L. lactis* subsp. *cremoris* are commonly used. In particular, the authors evaluated the impact of milk heat treatments and ripening temperatures on lactococcal starter and NSLAB throughout maturing of Cheddar cheese and the results showed that lactococci remained dominant throughout the ripening process.

The results presented in this study, however, do not shed light into the possible contribution of *L. lactis* in terms of organoleptic characteristics of the final cheese product. Thus, as future prospective, it will be important to investigate the role, in terms of metabolic activities, of this microorganism during cheese ripening. In particular, it will be interesting to understand which *L. lactis* functions are being carried out in each specific phase of the production, with the final aim of improving technological processes and cheese quality. A major improvement in our knowledge about the activity of this microorganism could be harnessed to control cheese ripening and the transfer of this knowledge to dairy industry could lead to the selection of new *L. lactis* strains with specific metabolic traits.
